# Innate Immunity in Fruit Flies: A Textbook Example of Genomic Recycling

**DOI:** 10.1371/journal.pbio.0020276

**Published:** 2004-08-17

**Authors:** Shubha Govind, Ross H Nehm

## Abstract

Drosophila serve as a wonderful model for studying aspects of innate immunity, i.e. the physical, cellular, and molecular features that provide the first lines of defense against infections in flies and man

Organisms of vastly differing morphologies, ecologies, and behaviors—such as fruit flies and humans—are now known to share a multitude of molecular, cellular, and developmental processes. Not only is there extensive similarity in the sequences of fly and human genes, but in addition, almost all of the proteins and major signal transduction pathways that control cell division and differentiation in mammals are also found in the fruitfly Drosophila melanogaster (Rubin et al. 2000; http://flybase.bio.indiana.edu/). Components in these pathways perform the same biochemical functions and act in the same order in both fruitfly and mammalian cells.

Evolutionary conservation is of considerable practical and theoretical importance to biologists. First, it provides a valuable source of data for the reconstruction of phylogeny (Salemi and Vandamme 2003). Evolutionary connections between organisms that were once hidden by morphology have now been exposed in genomic analyses. Second, the conservation of evolutionary processes or traits is a prime area of investigation in theoretical evolutionary biology (Gould 2002). What can, and cannot, be changed evolutionarily? In a constantly evolving world, how can any biological system or trait survive unchanged (Van Valen 1973)? Finally, conservation provides fundamental insights into how complex biological systems, such as immunity, are assembled, maintained, and altered in evolution.

## Elements of Immunity

“Innate” immunity refers to the variety of physical, cellular, and molecular features that provide the first lines of defense against infections. The relatively quick innate immune responses operate along with slower but more targeted adaptive immune responses that generate antigen-specific mechanisms that eventually lead to the destruction and elimination of the pathogen.

In mammals, the skin and the epithelial lining of the mucosal tissues act as the primary nonspecific barriers, impeding infectious agents from entering the body. The mucous membrane barrier traps microorganisms, and the cilia present on the epithelial cells assist in sweeping the microbes towards the external openings of the respiratory and gastrointestinal tracts. If infectious agents gain entry into the body, internal innate immune responses become activated and rapidly eliminate the infection. Internal innate immune agents and responses include (amongst others) low pH of the stomach and vagina, proteolytic enzymes and bile in the small intestine, and phagocytosis.

Phagocytosis is a fundamental innate immune mechanism carried out by a number of different cell types, including macrophages. Specific macrophage subpopulations are associated with different tissues (alveolar macrophages in the lung, microglial cells in the central nervous system, etc.). Their main function is to consume microorganisms, other foreign substances, and old, dying cells.

Innate immunity is present from birth, and the information for innate immune responses is inherited. Cells in the mammalian innate immune system (e.g., macrophages) detect “microbial nonself” by recognizing pathogen-associated molecular patterns (PAMPs; Janeway 1989). PAMPs are products of microbial metabolism that are conserved over evolution, distributed in a wide variety of pathogens, and not found in host cells. Lipopolysaccharide is an example of a PAMP and is found in bacteria, viruses, and fungi. Receptors, called pattern recognition receptors, are present on surfaces of host cells and recognize PAMPs. When activated, pattern recognition receptors induce intracellular signaling via the transcription factor NF-κB, resulting in the activation of genes involved in host defense.

Adaptive immunity is characterized by greater specificity than innate immunity, as the adaptive immune response can not only distinguish foreign cells from self, but can also distinguish one foreign antigen from another. Another hallmark of adaptive immunity is memory, which enables the body to remember specific adaptive responses in response to specific antigens. Immunological memory allows the body to make a greater and more rapid second response when the body is reinfected by the same pathogen. Immunological memory underlies both immunization and resistance to reinfection, conferring a tremendous evolutionary advantage to vertebrates. The adaptive immune response has nearly infinite flexibility: the T and B lymphocytes of the acquired immune system can rearrange the elements of their immunoglobulin and T-cell receptor genes to create billions of clones with distinct antigen receptors. In organisms where both innate and acquired immune systems are present, there is a clear interdependence between the two systems. For a fully functional immune system, these components must act in synergy.

## Innate Immunity in Drosophila

**Figure 1:**
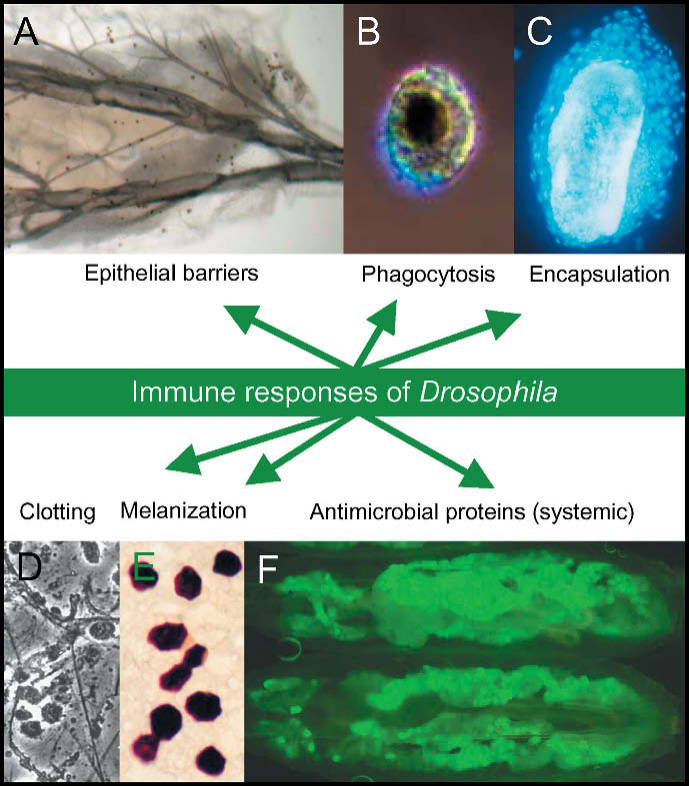
Innate Immune Responses of Drosophila (A) Posterior region of a third instar larva showing the cuticle and the trachea. These structures provide a physical barrier against infections. Cellular immune reactions consist of phagocytosis, encapsulation, and melanization. (B) A dead and melanized crystal cell phagocytosed by a plasmatocyte. (C) Encapsulation of an egg of a Drosophila parasite. The parasite is a wasp that normally infects larvae. Cells surrounding the egg are lamellocytes. The cells and the egg are stained with a fluorescent nuclear stain. (D) Clot formation occurs during wound healing. (E) Crystal cells in contact with the larval cuticle. The contents of the crystal cells are melanized. Melanization occurs in response to intruding pathogens or parasites and is also observed during wound healing. (F) Humoral immune reaction. The expression of antimicrobial peptides in the larval fat body is induced by microbes. Cells of the fat body appear green due to the presence of a transgene encoding the green fluorescent protein, under the control of the *drosomycin* promoter. The *drosomycin* promoter is activated in response to fungal infections and is under the control of the Toll pathway (see Figure 2). Antimicrobial peptides are released from the fat body into the hemolymph. This response is therefore systemic. A similar antimicrobial gene activation response can occur locally in specific body parts such as the trachea or the gut (not shown).

Because it lacks an adaptive immune response, Drosophila melanogaster serves as a wonderful model for studying aspects of the innate immune system that might otherwise be obscured by the actions of the adaptive immune response. Insects defend themselves against parasites and pathogens by invoking a multitude of innate immune responses (Figure 1; for more details, see recent reviews by Hoffmann and Reichhart [2002], Hultmark [2003], Brennan and Anderson [2004], Meister [2004], and Theopold et al. [2004]). Like humans, Drosophila protects itself against microbes and parasites via epithelial barriers: for example, epithelial cells of the trachea, gut, genital tract, and Malpighian tubules produce antimicrobial peptides (local response).

Once within the body cavity, microbes may be consumed by the phagocytic blood cells called plasmatocytes (Figure 1). Larger pathogens (such as eggs of parasitic wasps) are inactivated by encapsulation, an immune response carried out by specialized cells called lamellocytes (Figure 1). Lamellocytes differentiate in response to macroscopic pathogens, and their precursors are thought to reside in the larval lymph gland. The transcription factors (GATA, Friend-of-GATA, and Runx family proteins) and signal transduction pathways (Toll/NF-κB, Serrate/Notch, and JAK/STAT) that are required for specification and proliferation of blood cells during normal hematopoiesis, as well as during the hematopoietic proliferation that accompanies immune challenge, are conserved (Evans et al. 2003; Meister 2004). In this issue of *PLoS Biology*, Crozatier et al. (2004) identify the transcription factor Collier as being critical for the differentiation of lamellocytes in Drosophila. The mammalian ortholog of Collier (Early B-cell Factor) is involved in B-cell differentiation in mice.

In addition to triggering cellular immune responses, invading pathogens also activate humoral reactions. Microbes induce the rapid secretion of antimicrobial peptides from the cells of the fat body into the larval or adult body cavity (systemic response; Figure 1). A microbial infection initiates a zymogen cascade that plays a crucial role in the activation of the antimicrobial genes in the fat body. Infection or wounding also triggers a protein-cleaving cascade that results in the production of toxic intermediates and melanin around microbes or wound sites. This proteolytic cascade is similar to the vertebrate clotting cascade. Drosophila hemolymph also coagulates and participates in host defense and wound healing (Figure 1; Theopold et al. 2004). Given the evolutionary success of insects, this combination of defense mechanisms has proven to be extremely effective, allowing insects to thrive in septic environments.

## NF-κB Activation: The Toll and Imd Pathways of Drosophila

**Figure 2:**
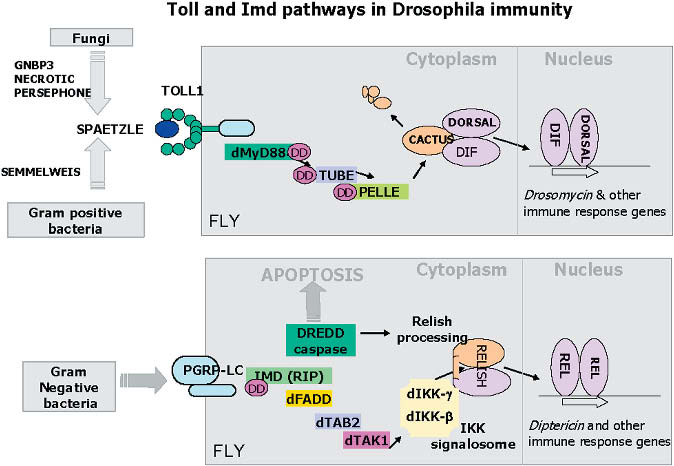
Molecular Components of the Toll and Imd Pathways Involved in Drosophila Immunity Toll is activated by the processed Spätzle (left). Toll activation leads to intracellular signaling via cytoplasmic proteins Tube and Pelle, leading to the degradation of Cactus and nuclear localization of NF-κB proteins Dorsal and Dif. These transcription factors bind to promoters of target genes, such as *drosomycin*, activating their transcription. The NF-κB protein for the Imd pathway, Relish, activates *diptericin* transcription. The signaling events resulting in Dorsal/Dif/Relish activation in the fly are “recycled” in mammals in the activation of mammalian NF-κB. See reviews and De Gregorio et al. (2002) for more details.

The Drosophila genome encodes several members of the multifunctional Toll family of receptors (Beutler and Rehli 2002). Mutations in the Drosophila Toll gene (as well as in other components in the pathway) make the fly susceptible to fungal or gram-positive bacterial infections. However, Toll does not act as a pattern recognition receptor in the fly; instead its activation depends on the presence of the processed (active) form of the growth-factor-like polypeptide Spätzle. Processing of Spätzle depends on a serpin-controlled proteolytic cascade (Figure 2).

While components of the Drosophila Toll pathway were identified in earlier genetic screens for developmental mutants, those in the Imd pathway have been the focus of more recent studies, mainly in the context of Drosophila immunity (Hoffmann and Reichhart 2002; Hultmark 2003). The effector NF-κB transcription factor of the Imd pathway is Relish, which upon immune activation is cleaved by the Dredd caspase (Figure 2). Using a combination of the RNA interference approach of silencing gene function and a high-throughput cell culture assay, Foley and O'Farrell (2004) report the identification of two new conserved members of this Imd pathway: Sickie is a novel protein required for Relish activation, and Defense repressor 1 is a novel inhibitor of the Dredd caspase.

The impressive progress in our understanding of innate immunity in Drosophila is now guiding scientists to explore the immune system of other insects such as the mosquito, Anopheles gambiae, that spreads human malaria. Immune responses in this mosquito are linked to the elimination of the malarial parasites (Osta et al. 2004). A comparison of the immunity-related genes in Anopheles and Drosophila reveals the presence of the Toll signaling pathway in the mosquito genome, even though there are some differences in genes encoding pathogen recognition and signal transduction molecules (Christophides et al. 2002). A detailed and comparative view of the genetic mechanisms underlying their host defense will contribute to the identification of new targets for insecticide development, and provide opportunities for controlling the transmission of pathogens.

## Concluding Remarks

The homologs of many genes involved in innate immune responses in flies and humans have also been found in mice, sharks, nematodes, and plants (e.g., Pujol et al. 2001; Nurnberger and Brunner 2002). In species studied to date, host defense appears to be mediated by homologous proteins. Taken together, these findings suggest that the regulatory mechanisms of host defense may be hard-wired in the genome much as DNA replication and cell division are. Protein motifs, domains, and signaling elements have, for millions of years, not only retained their ancestral biochemical features but have also continued to participate in similar physiological responses. It is crucial that our evolving knowledge of “genomic recycling” be used to enhance our understanding of the evolution of humans, not only in the context of “descendants of ancient apes,” but in the larger context of our fundamental unity and shared genetic history with all other species. This simple but fundamental idea has yet to be adopted by the majority of our students and teachers. Unless we do more to overcome resistance to the idea that humans share deep evolutionary connections with all animal life, students will become increasingly isolated from an understanding of, and participation in, the genomics and bioinformatics revolution that is transforming the biological and biomedical sciences.

## Abbreviation

PAMP: pathogen-associated molecular pattern
